# Crosstalk between hnRNP K and SET in ATRA‐induced differentiation in acute promyelocytic leukemia

**DOI:** 10.1002/2211-5463.13210

**Published:** 2021-06-17

**Authors:** Karina Stringhetta Padovani, Renata Nishida Goto, Lais Brigliadori Fugio, Cristiana Bernadelli Garcia, Vani Maria Alves, Maria Sol Brassesco, Lewis Joel Greene, Eduardo Magalhães Rego, Andréia Machado Leopoldino

**Affiliations:** ^1^ Department of Clinical Analyses, Toxicology and Food Sciences School of Pharmaceutical Sciences of Ribeirão Preto University of São Paulo Brazil; ^2^ CEPID‐FAPESP Center for Cell Based Therapy Regional Blood Center of Ribeirão Preto Brazil; ^3^ Department of Cellular and Molecular Biology and Pathogenic Bioagents School of Medicine of Ribeirão Preto‐FMRP University of São Paulo Ribeirão Preto Brazil; ^4^ Department of Biology Faculty of Philosophy, Sciences and Letters of Ribeirão Preto University of São Paulo Brazil; ^5^ Department of Internal Medicine School of Medicine of Ribeirão Preto‐FMRP University of São Paulo Ribeirão Preto Brazil

**Keywords:** APL, ATRA, ERK, hnRNP K, promyelocyte differentiation, SET

## Abstract

HnRNP K protein is a heterogeneous nuclear ribonucleoprotein which has been proposed to be involved in the leukemogenesis of acute promyelocytic leukemia (APL), as well as in differentiation induced by all‐trans retinoic acid (ATRA). We previously demonstrated a connection between SET and hnRNP K function in head and neck squamous cell carcinoma (HNSCC) cells related to splicing processing. The objective of this study was to characterize the participation of hnRNP K and SET proteins in ATRA‐induced differentiation in APL. We observed higher (5‐ to 40‐fold) levels of hnRNP K and SET mRNA in APL patients at the diagnosis phase compared with induction and maintenance phases. hnRNP K knockdown using short‐hairpin RNA led to cell death in ATRA‐sensitive NB4 and resistant NB4‐R2 cells by apoptosis with SET cleavage. In addition, hnRNP K knockdown increased granulocytic differentiation in APL cells, mainly in NB4‐R2 with ATRA. hnRNP K knockdown had an effect similar to that of treatment with U0126 (an meiosis‐specific serine/threonine protein kinase/ERK inhibitor), mainly in NB4‐R2 cells. SET knockdown in APL cells revealed that apoptosis induction in cells with hnRNP K knockdown occurred by SET cleavage rather than by reduction in SET protein. Transplantation of NB4‐R2 cells into nude mice confirmed that arsenic trioxide (ATO) combined with U0126 has higher potential against tumor progression when compared to ATO. Therefore, hnRNP K/SET and ERK are potential therapeutic targets for both antineoplastic leukemia therapy and relapsed APL patients with ATRA resistance.

## Graphical abstract

ERK inhibition or heterogeneous nuclear ribonucleoprotein K knockdown decreases cell viability, increases all‐trans retinoic acid (ATRA)‐induced differentiation in acute promyelocytic leukemia (APL) ATRA‐resistant cells, and promotes SET cleavage. Furthermore, findings obtained using a xenograft NB4‐R2 tumor model suggest the use of U0126, a meiosis‐specific serine/threonine protein kinase/ERK inhibitor, combined with arsenic trioxide as a potential therapeutic strategy to be explored for APL. Our study highlights crosstalk between ERK/hnRNPK/SET in APL and ATRA‐induced differentiation.

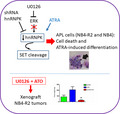

**Abbreviations**
APL: acute promyelocytic leukemia
ATO: arsenic trioxide
ATRA: all‐trans retinoic acid
Bcl‐xl: Bcl‐2‐like protein 1
BM: bone marrow
C/EBPα: CCAAT enhancer‐binding protein alpha
CML: chronic myeloid leukemia
DMSO: dimethyl sulfoxide
DP: diagnosis phase
ERK1/2: extracellular signal‐regulated kinases ½
HDAC1: histone deacetylase 1
hnRNP K: heterogeneous nuclear ribonucleoprotein K
HNSCC: head and neck squamous cell carcinoma
MAPK: mitogen‐activated protein kinase
MEK 1/2: meiosis‐specific serine/threonine protein kinase ½
MP: maintenance phase
NC: negative control
PIP: postinduction
PML‐RARA: promyelocytic leukemia‐retinoic acid receptor alpha genes
PP2A: phosphatase 2A subunit C
PU.1: hematopoietic transcription factor PU.1
qRT‐PCR: quantitative real‐time PCR
SET: SET nuclear proto‐oncogene
sh_K: short‐hairpin RNA for hnRNP K
shRNA: short‐hairpin RNA interference

HnRNP K protein is a heterogeneous nuclear ribonucleoprotein that acts as a transcriptional regulator by binding directly to DNA and RNA through the homologous domain K, which acts as a nucleic acid‐regulated docking platform [1, 2]. This protein, present in the nucleus, cytoplasm, and mitochondria, is involved in the regulation of gene expression, acting on transcription, RNA shuttling, RNA splicing, and translation processes [1]. Furthermore, hnRNP K has been considered as an prognostic marker in various types of cancer, including colorectal cancer [3], HNSCC [4], and chronic myeloid leukemia (CML) [5, 6]. Besides that, its involvement in the leukemogenesis of acute promyelocytic leukemia (APL) has been proposed, as well as in the differentiation induced by all‐trans retinoic acid (ATRA) [7].

Extracellular signal‐regulated kinases (ERK1/2 proteins) belong to the mitogen‐activated protein kinase (MAPK) family and regulate the activation of several cellular processes such as proliferation, cell differentiation, and survival, upon activation by numerous mitogen ligands. As MAPK pathway often presents deregulated in cancer, the components of this cascade have been proposed as targets in antineoplastic therapy [8]. The interaction of the hnRNP K protein with other proteins is also regulated via phosphorylation by MAPK/ERK pathway [9]. ERK inhibitor has demonstrated effect on the decrease in hnRNP K levels in CML, suggesting an antitumoral potential [10].

Recently, our group has demonstrated a connection between SET and hnRNP K function in HNSCC cells related to splicing processing [11]. The SET gene was originally identified as a component of the SET‐CAN fusion gene in acute undifferentiated leukemia. SET, a 39 kDa protein, is known to be an endogenous inhibitor of phosphatase 2A subunit C (PP2A) [12, 13]. However, SET is a multifunctional protein that participates in the response to oxidative stress [14], autophagy [15], DNA damage repair [16], apoptosis [17], cell migration [18], cell cycle control [19], and chromatin remodeling [20]. SET overexpression, reducing PP2A activity, has been reported and associated with a poor prognosis in chronic lymphocytic leukemia of B [21], CML [22], AML [23] cells, and other types of cancer [15, 24].

In APL, the oncogene promyelocytic leukemia‐retinoic acid receptor alpha genes (PML‐RARA), resulting from reciprocal translocation t(15,17), leads to the compromise of granulocytic differentiation and genetic stability by directly interfering in the transcriptional regulation of RARA and PML, respectively [25]. ATRA promotes granulocytic differentiation restoring transcriptional activation of RARA target genes that are involved in cellular differentiation and by PML‐RARα degradation, but the isolated therapy with ATRA leads to transient remission of disease [26], suggesting involvement of other mechanisms in the self‐renewal of APL cells. Other molecular variables such as CCAAT enhancer‐binding protein alpha (C/EBPα) and MYC, for example, onset of an occurrence of PML‐RAR, have already been demonstrated in APL and associated with the leukemogenesis of this type of leukemia. The combination of ATRA with anthracycline chemotherapy is the mainstay treatment in some cases for APL, which promotes complete remission in 90% of cases and long‐term disease‐free status in 85% [27, 28]. However, the differentiation syndrome [29, 30] and resistance to ATRA [31, 32] observed in patients interfere with the treatment. Thus, additional searches for new therapeutic targets are required.

After the demonstration that hnRNP K and SET are overexpressed in the APL patients compared with controls, in the present study we knocked down these proteins in both ATRA‐sensitive (NB4) and ATRA‐resistant (NB4‐R2) cell lines by short‐hairpin RNA interference (shRNA) and assessed their involvement in ATRA‐induced cell differentiation, as well as their contributions to the maintenance and survival of leukemic cells. Besides, we showed the potential of an ERK inhibitor as a putative APL chemotherapy combined with arsenic trioxide (ATO).

## Results

### *HNRNPK* and *SET* mRNAs in APL patients

**Fig. 1:**
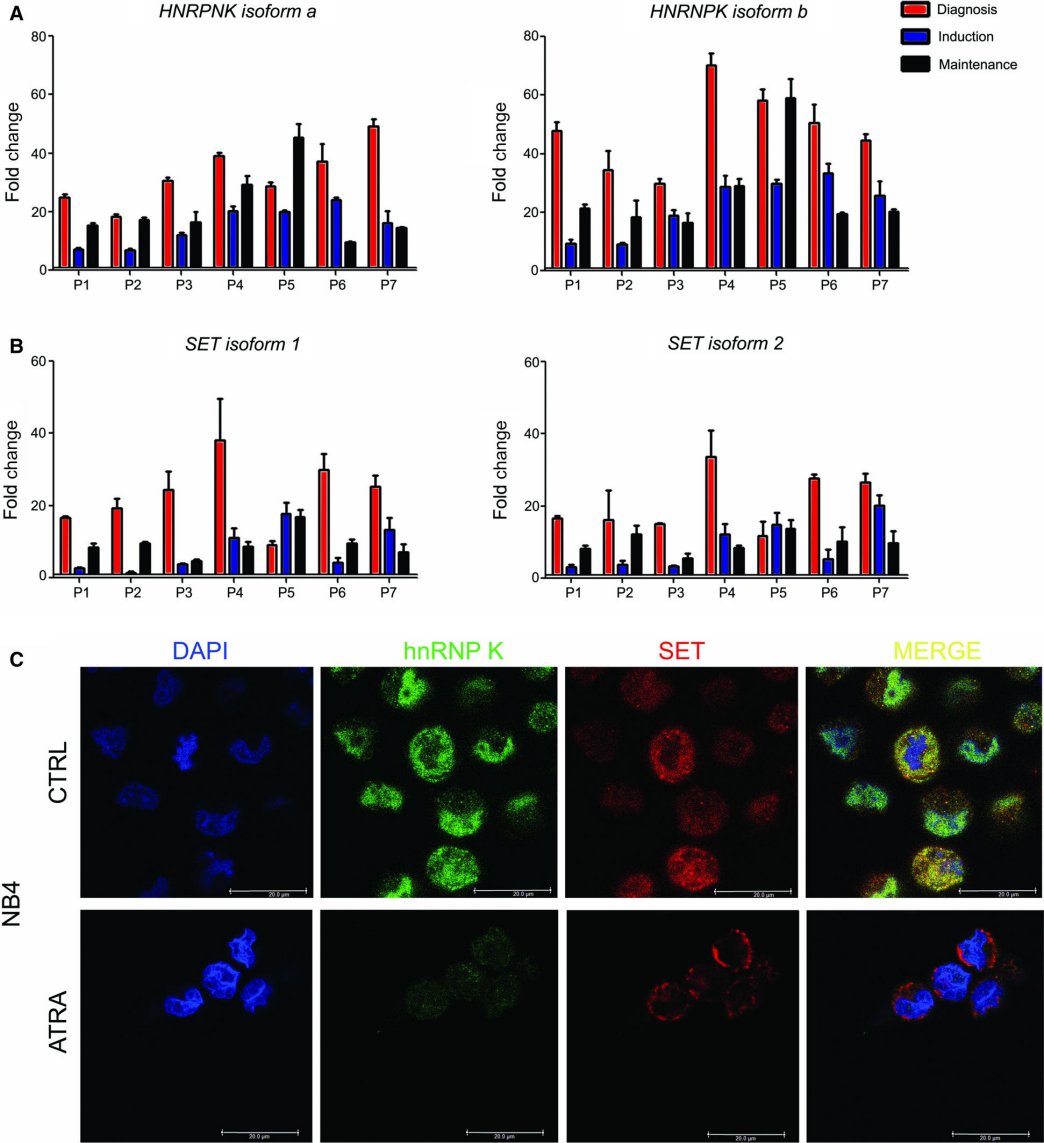
*HnRNP K* and *SET* were overexpressed in the BM of APL patients compared with BM of healthy individuals. (A) The gene expression of *HNRNPK* and (B) *SET* was determined by qRT‐PCR in samples of patients (*n* = 7) during DP of APL, induction (PIP) and MP of treatment, compared with BM samples from healthy individuals (*n* = 2). Relative mRNA expression levels were calculated by the 2^−ΔΔCT^ method; β‐globin was used as the housekeeping gene. Data are reported as fold change in each phase (DP/IP/MP) compared with BM. Error bars represent SD of experiments performed in triplicate. (C) Confocal microscopy showing hnRNP K (green) and SET (red) proteins staining before (CTRL) and after ATRA treatment in NB4 cells. Nuclei were stained by DAPI (blue). Fluorescence overlapping is shown in yellow. Scale bar indicates 20 µm.

*HNRNPK* and *SET* mRNAs were measured by quantitative real‐time PCR (qRT‐PCR) in bone marrow (BM) samples from APL patients. Two transcripts, each encoding isoform a and b proteins of each gene, were analyzed. mRNA levels were compared at diagnosis (DP), postinduction (PIP), and maintenance phase (MP) for each patient. The hnRNP K and SET mRNAs were expressed 5‐ to 40‐fold higher in DP compared with PIP and MP (Fig. 1A,B). Patient 5 (P5) suffered a relapse during MP of treatment, which was accompanied by an increase in the expression of both genes, mainly *HNRNPK* (Fig. 1A,B). The data suggest a relationship between the levels of both genes in the APL leukemogenesis as well as during the treatment of the same patient.

**Fig. 2:**
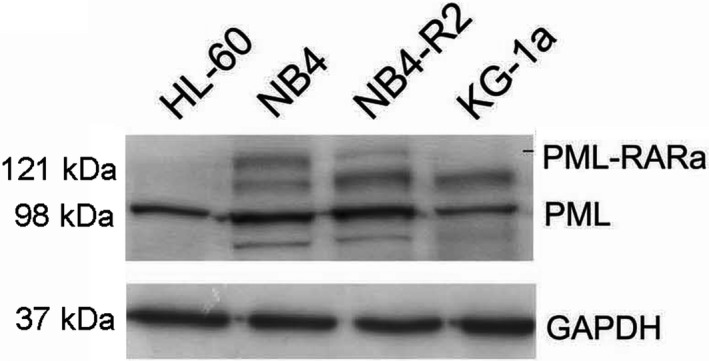
NB4 and NB4‐R2 cells are PML‐RARa positive. The PML and PML‐RARa protein profiles were analyzed in HL‐60, NB4, NB4‐R2, and KG‐1a cells by immunoblotting using anti‐PML. GAPDH was used as a loading control.

Next, we analyzed the distribution of SET and hnRNP K proteins in NB4 cells (ATRA‐sensitive cell line) by using immunofluorescence assay and microscopy (Fig. 1C). The colocalization of the proteins was lost when the cells were incubated with ATRA. Besides, this was accompanied by a slight reduction in SET protein and none apparent alteration in hnRNP K protein level when we analyzed by western blot (as shown in Fig. 5C). It reinforced our results in patient samples and our hypothesis on a potential crosstalk between hnRNP K and SET in ATRA‐induced differentiation; consequently, their deregulations may have implications in ATRA resistance mechanisms. In addition, there are studies reporting each protein individually in leukemia [4, 5, 33, 34]. Then, we selected the ATRA‐sensitive cell line NB4 and the ATRA‐resistant cell line NB4‐R2, which are PML‐RAR fusion gene positive (Fig. 2), as a model for our study.

### HnRNP K is required for the survival and maintenance of leukemic cells

**Fig. 3:**
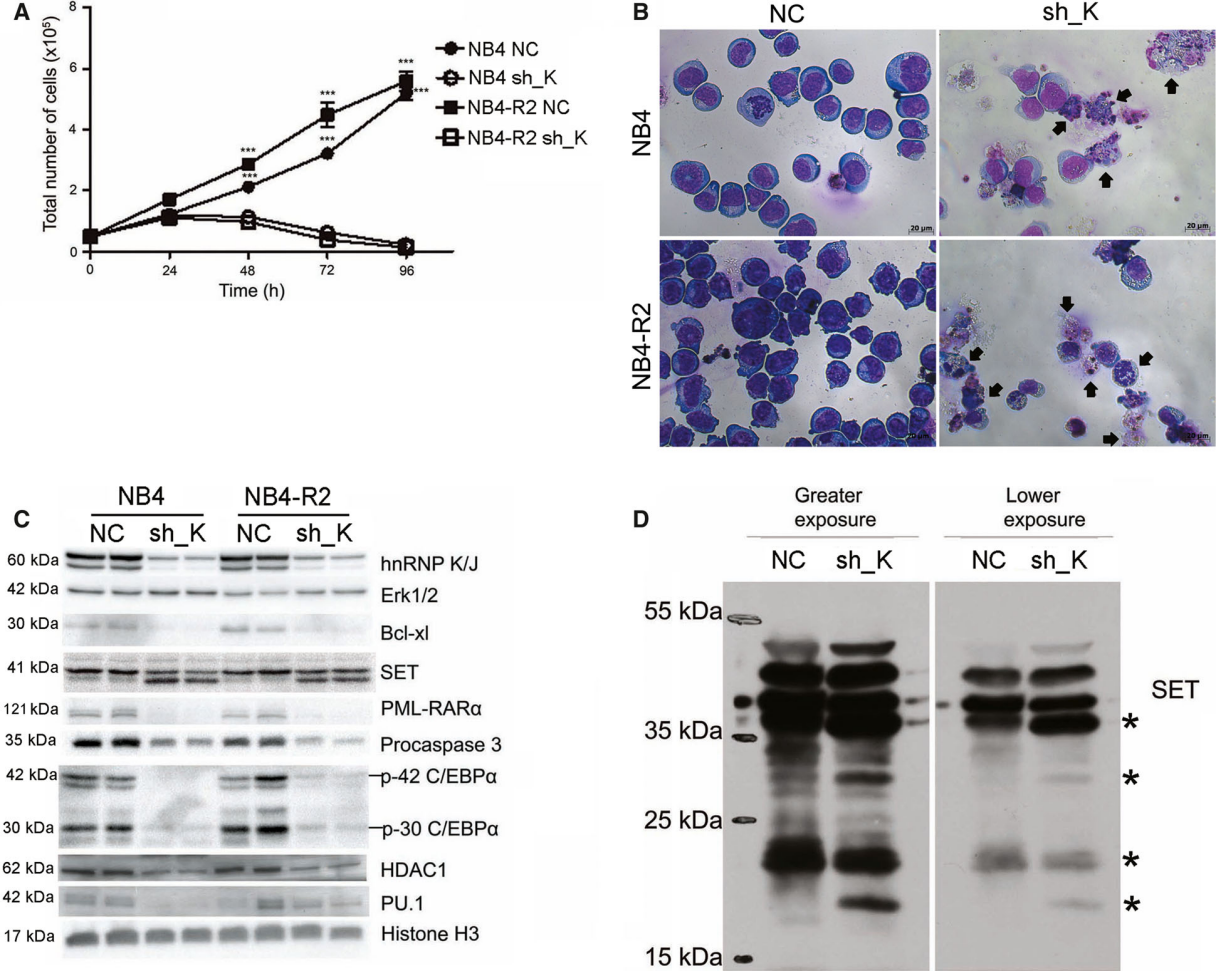
HnRNP K knockdown leads to cell proliferation reduction that is accompanied by apoptosis, PML‐RARα degradation, and SET protein cleavage. Cells were transduced with sh_K for hnRNP K or a shRNA‐NC, and maintained under puromycin (0.5 µg·mL^−1^) selection. (A) For the growth curve, 5 × 10^4^ cells·mL^−1^ were seeded into a 24‐well plate in triplicate, and the count was performed every 24 h for 96 h, using the Trypan Blue exclusion method. Data are presented as mean ± SD of two independent experiments performed in triplicate. Statistical analysis was performed by one‐way ANOVA followed by the Bonferroni post‐test, ***(*P* < 0.001). (B) Cells stained by panoptic showed apoptotic vesicles in sh_K cells (black arrows) at 400× magnification under a brightfield microscope. Scale bars indicate 20 µm. (C) After 72 h from puromycin selection, cells were recovered and the protein extraction performed. Immunoblotting analysis confirmed the decrease in the levels of hnRNP K and showed reduction in Bcl‐xl (anti‐apoptotic), PML‐RARα, procaspase‐3, C/EBPα (p42 and p30), HDAC1, and PU.1. Furthermore, hnRNP K knockdown led to SET cleavage and increased ERK protein levels. Histone H3 was used as a loading control. (D) Another immunoblotting using a monoclonal antibody for SET confirmed an increase in SET cleavage levels in NB4 cells with hnRNP K knockdown. The asterisk means cleaved SET protein bands.

Initially, we confirmed that hnRNP K is an essential protein for APL cell survival and proliferation by using short‐hairpin RNA for hnRNP K (sh_K; Fig. 3A). Besides, microscopic analysis showed the presence of apoptotic bodies in both NB4 and NB4‐R2 cells with hnRNP K knockdown (Fig. 3B). Accordingly, we analyzed proteins related to survival [procaspase‐3 and Bcl‐2‐like protein 1 (Bcl‐xl)], differentiation (PML‐RARα, ERK1/2, PU.1, C/EBPα), and hnRNP K function [SET, histone deacetylase 1 (HDAC1)]. Interestingly, a reduced level of the procaspase‐3 and Bcl‐xl proteins was accompanied by SET cleavage in APL sh_K cells, suggesting cell death by apoptosis. Notably, ERK1/2 levels were increased while the levels of PML‐RARα (oncoprotein), HDAC1, PU.1, and C/EBPα (isoforms p42 and p30 kDa) were decreased (Fig. 3C). The increase in SET cleavage (bands below 39 kDa) in sh_K cells was confirmed using a monoclonal antibody against SET (Sigma‐Aldrich Inc., Saint Louis, MO, USA). Besides, the antibody recognized other cleaved SET forms, namely ~ 35, ~ 28, ~ 20, and ~ 18 kDa (Fig. 3D). These results suggest that hnRNP K levels in NB4 and NB4‐R2 cell lines are directly correlated with cell survival and differentiation signaling.

**Fig. 4:**
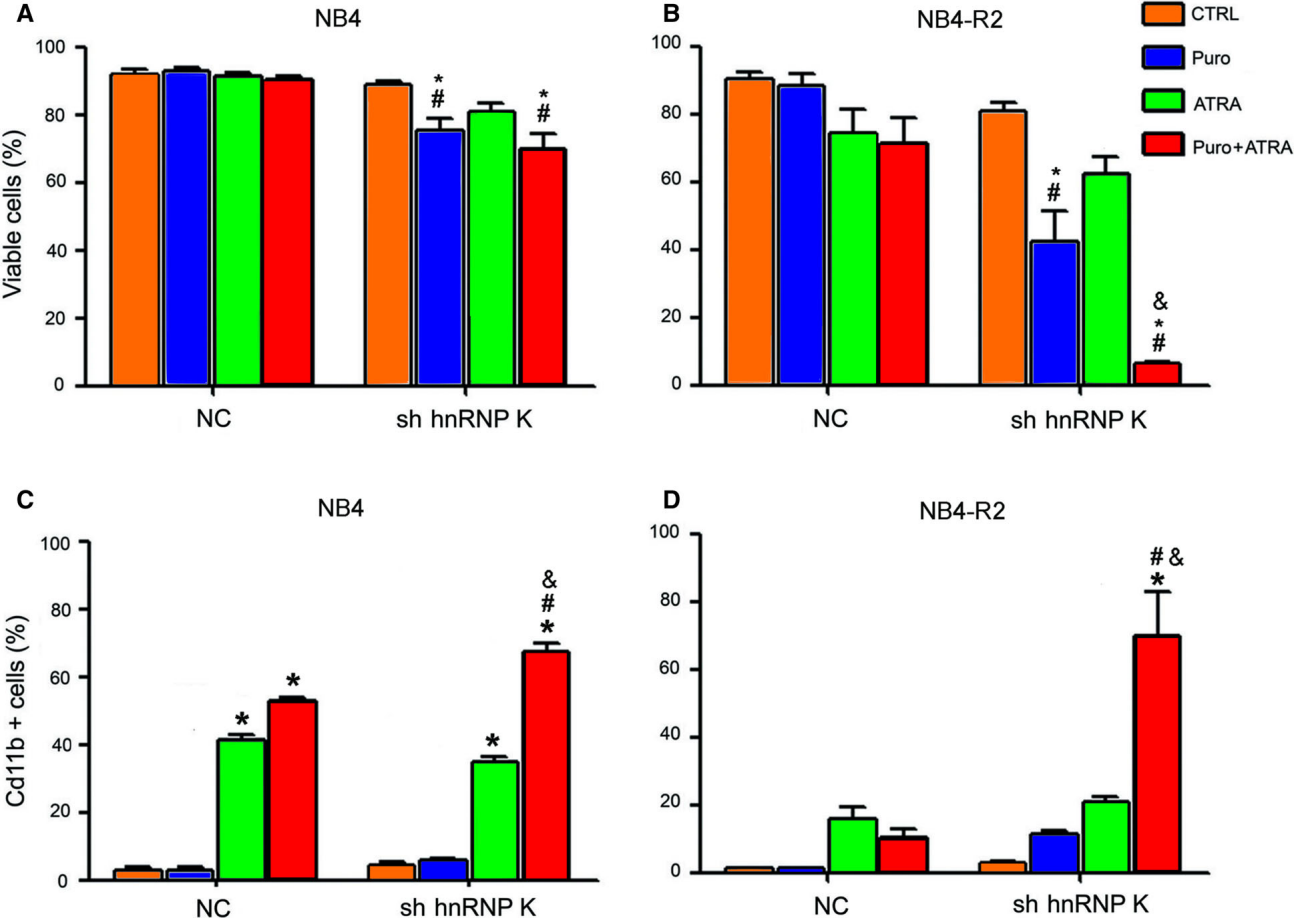
HnRNP K knockdown leads to the loss of NB4 and NB4‐R2 cell viability, favors cell differentiation, and sensitizes ATRA‐resistant cells. (A) NB4 and (B) NB4‐R2 cells were transduced with short‐hairpin RNA to hnRNP K. The selection of the cells after transduction was performed with 0.15 µg·mL^−1^ puromycin for NB4 cells and 0.2 µg·mL^−1^ for NB4‐R2. The reduction in hnRNP K was confirmed by immunoblotting using anti‐hnRNP K. ATRA (1 µm) treatment was performed as previously described. After selection with puromycin for 96 h, cell viability assays were performed by the PI incorporation method and analyzed by flow cytometry. (C) NB4 and (D) NB4‐R2 (1 × 10^5^) cells were incubated with anti‐CD11b PE, and cellular differentiation was analyzed by flow cytometry as the percentage of CD11b positive cells. Data are presented as mean ± SD of two independent experiments performed in triplicate. CTRL = control. NC, short‐hairpin RNA‐NC. Statistical analysis was performed by one‐way ANOVA followed by the Bonferroni post‐test, ^#^(*P* < 0.001) NC compared with sh hnRNPK, and *(*P* < 0.001) compared with the control (CTRL); ^&^(*P* < 0.001) Puro+ATRA compared with ATRA.

Initially, we selected NB4 and NB4‐R2 cells with the shRNA‐negative control (NC) and shRNA for hnRNP K (sh_K) with 0.5 µg·mL^−1^ puromycin, but the hnRNP K knocked down cells were lost within a few days. Then, a cell growth curve was obtained after transduction under different puromycin concentrations for both cell lines to establish the ideal conditions for hnRNP K knockdown with enough number of live cells (data not shown). The concentrations of puromycin selected were 0.15 µg·mL^−1^ for NB4 and 0.2 µg·mL^−1^ for NB4‐R2 (Fig. 4A). hnRNP K knockdown (sh_K), combined with ATRA treatment, promoted a higher loss of viability in NB4‐R2 cells than in NB4 cells (Fig. 4B). Of note, cell death observed in sh_K cells was followed by increased cell differentiation, as assessed by CD11b labeling (Fig. 4C,D). These results provide evidence that the reduction in hnRNP K levels can modify the status of the NB4‐R2 cell line regarding ATRA‐sensitive behavior. They indicate that hnRNP K is an important protein involved in the resistance to ATRA‐induced differentiation and APL cell survival.

### MEK inhibitor reduces hnRNP K protein levels and mimics hnRNP K knockdown in APL cell lines

**Fig. 5:**
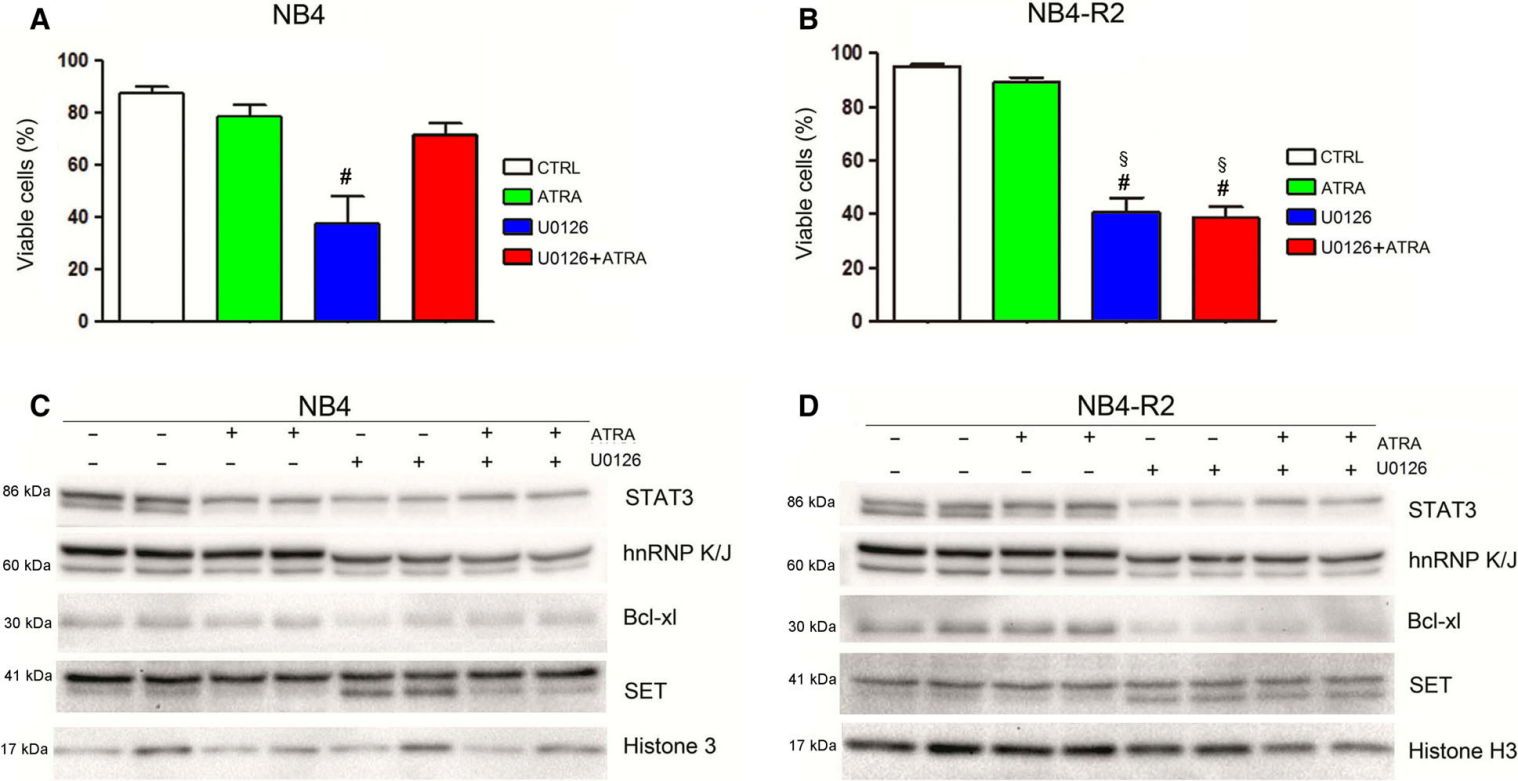
Inhibition of MAPK signaling led to effects similar to those observed with hnRNP K knockdown in APL cell lines. NB4 and NB4‐R2 cells (2 × 10^5^ cells·mL^−1^ in six‐well plates performed in duplicate) were treated with 10 µm U0126 for 72 h, in association or not with ATRA, which was added 2 h after U0126. Cell viability assay in NB4 (A) and NB4‐R2 (B) was performed by the PI incorporation method. Cells were incubated with 1 µg·mL^−1^ PI immediately before reading and analyzed by flow cytometry. Data are reported as mean ± SD of three independent experiments performed in duplicate. Statistical analysis was performed by one‐way ANOVA (Kruskal–Wallis test and Dunn post‐test), ^#^compared with control (CTRL) and ^§^compared to ATRA with *P* < 0.05. (C) Immunoblotting analysis revealed that U0126 treatment of both cell lines, NB4 and (D) NB4‐R2, associated or not with ATRA decreased hnRNP K and Bcl‐xl levels and increased SET cleavage (mainly in NB4‐R2). STAT3 was reduced by U0126 and included as a MAPK/ERK downstream target‐like control of the experiment. Histone H3 was used as a loading control.

To assess a relationship between the ERK signaling and hnRNP K functions in APL, we used U0126, a MEK1/MEK2 inhibitor. The treatment with U0126 promoted a significant loss of cell viability in both APL cells (Fig. 5A,B). However, NB4 cells under ATRA + U0126 presented higher viability than NB4‐R2, suggesting a potential U0126 sensitivity associated with an undifferentiated APL behavior. Both NB4 and NB4‐R2 cell lines presented the same profile regarding hnRNP K protein levels, that is, a decrease in hnRNP K in the presence of the inhibitor (Fig. 5C,D). Furthermore, for both cells, Bcl‐xl levels were decreased and SET was cleaved in the presence of either U0126 or U0126 associated with ATRA, as observed in sh_K cells. These data indicate that the effect of hnRNP K protein knockdown could be mimicked by blocking MEK/ERK signaling, since hnRNP K phosphorylation is regulated by MEK/ERK. The STAT3 protein, a downstream target by ERK, was reduced in the presence of U0126 and ATRA (Fig. 5C,D). It suggests that NB4‐R2 (ATRA‐resistant cells/undifferentiated cells) is dependent on hnRNP K protein/ERK signaling to survive. It is supported by our result showing a reduced NB4 cell viability under U0126 (alone) but its recovery under ATRA + U0126.

### MEK inhibitor, in combination with arsenic trioxide therapy, leads to smaller tumors in a human NB4‐R2 xenograft murine tumor model

**Fig. 6:**
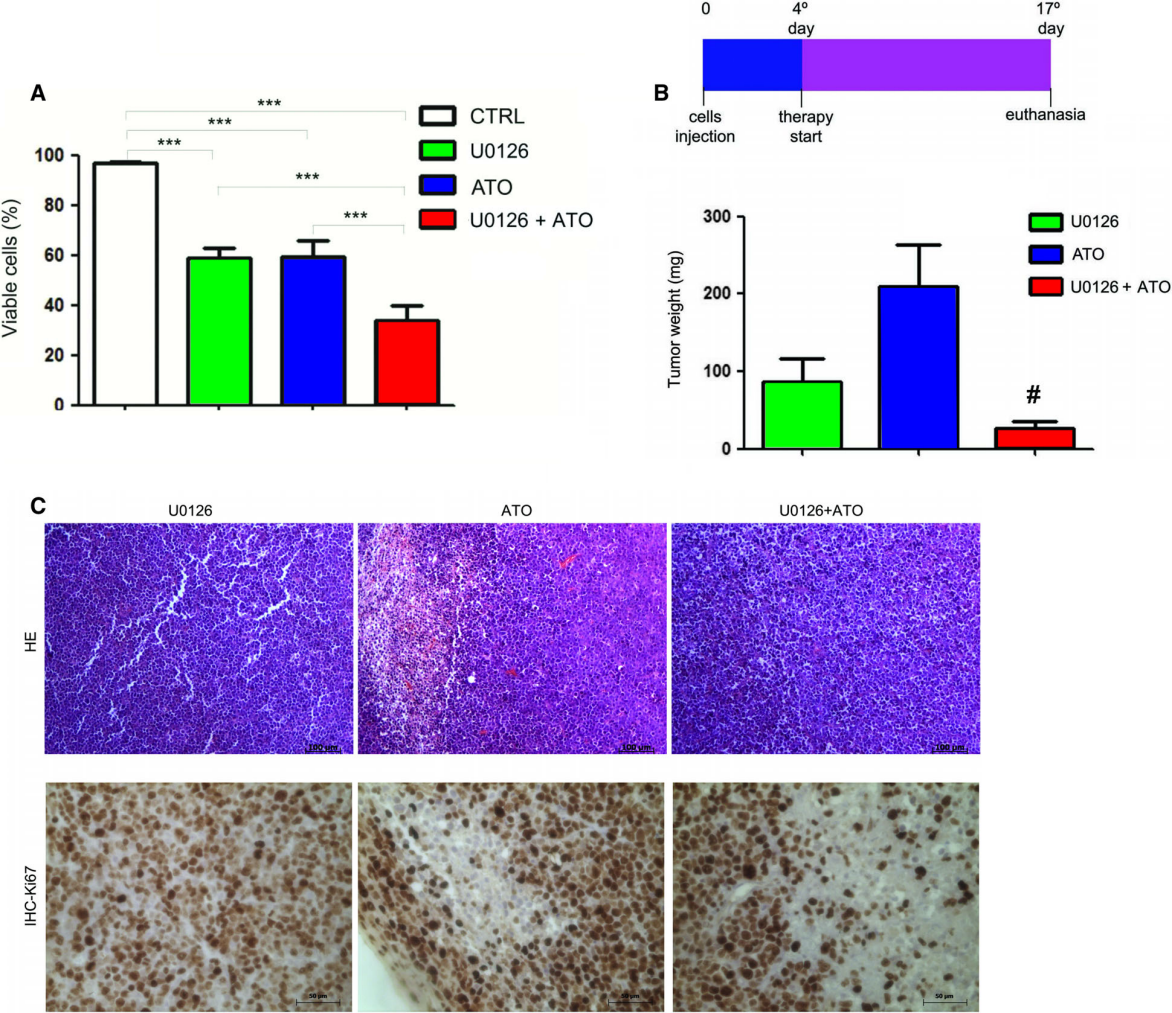
*In vivo* effect of U0126 in association or not with ATO using a NB4‐R2 xenograft murine tumor model. (A) NB4‐R2 cells (2 × 10^5^ cells·mL^−1^ in six‐well plates performed in duplicate) were incubated with 10 µm U0126 and 1 µm ATO for 96 h, combined, and isolated (CTRL). Cell viability assay in NB4‐R2 was performed by the PI incorporation method. Cells were incubated with 1 µg·mL^−1^ PI immediately before reading and analyzed by flow cytometry (BD FACSCalibur). Data are reported as mean ± SD of three independent experiments performed in duplicate. Statistical analysis was performed by one‐way ANOVA (one‐dimensional analysis of variance followed by the Bonferroni post‐test), ***(*P* < 0.001). (B) NB4‐R2 cells (5 × 10^6^) were transplanted in 8‐week‐old Balb/C male nude mice on the back of the animal bilaterally. Animals were randomly divided into groups: U0126 (*n* = 5, U0126 2.5 mg·kg^−1^), ATO (*n* = 5, 2.5 mg·kg^−1^), and U0126 + ATO (*n* = 5, U0126 2.5 mg·kg^−1^ + ATO 2.5 mg·kg^−1^). Animals in each group were identified from 1 to 5 by labeling the ear. Treatment was started on the fourth day after cell transplantation and performed every 48 h for a total of six applications. Animals were euthanized on the 17th day of the experiment. At autopsy, tumors were excised and weighed; the results are reported in mg. Data are reported as mean ± SD. Statistical analysis was performed by one‐way ANOVA followed by the Bonferroni post‐test, ^#^(*P* < 0.05). (C) H&E staining of tissues indicated hemorrhagic and necrosis areas. Immunohistochemistry (IHC) was performed using an anti‐Ki67, and the complexes were visualized by 3, 3‐diaminobenzidine staining. The tissue samples were counterstained by Harry's hematoxylin and analyzed under a light field microscope. Scale bars indicate 100 µm (HE) and 50 µm (IHC).

When the effect of ATO, a clinical approved treatment, was compared to U0126 and U0126 combined with ATO in the viability of NB4‐R2 cells *in vitro*, there was higher cell death in cells exposed to U0126 + ATO (Fig. 6A). In view of the positive results obtained with ATO plus U0126 in NB4‐R2 cells, an NB4‐R2 xenograft tumor model was used. The groups were as follows: U0126 (*n* = 5, U0126 2.5 mg·kg^−1^), ATO (*n* = 5, 2.5 mg·kg^−1^), and U0126 + ATO groups (*n* = 5, U0126 2.5 mg·kg^−1^ + ATO 2.5 mg·kg^−1^). The treatment started 4 days after cell transplantation every 48 h. Animals were euthanized on the 17th day of the experiment, and tumors were extracted and weighed (Fig. 6B). NB4‐R2 xenograft tumors from nude mice treated with the U0126 + ATO combination were smaller (Fig. 6B) than those receiving monotherapy, indicating that the combined treatment was more efficient. Histological analysis of tumor tissues after hematoxylin and eosin (H&E) staining showed that the tumors were extremely hemorrhagic, and the immunohistochemistry for Ki67, a proliferation marker, showed area with low proliferation (without Ki67 staining) and cell death (necrosis; Fig. 6C).

### SET knockdown facilitates cell differentiation in ATRA‐resistant cells

**Fig. 7:**
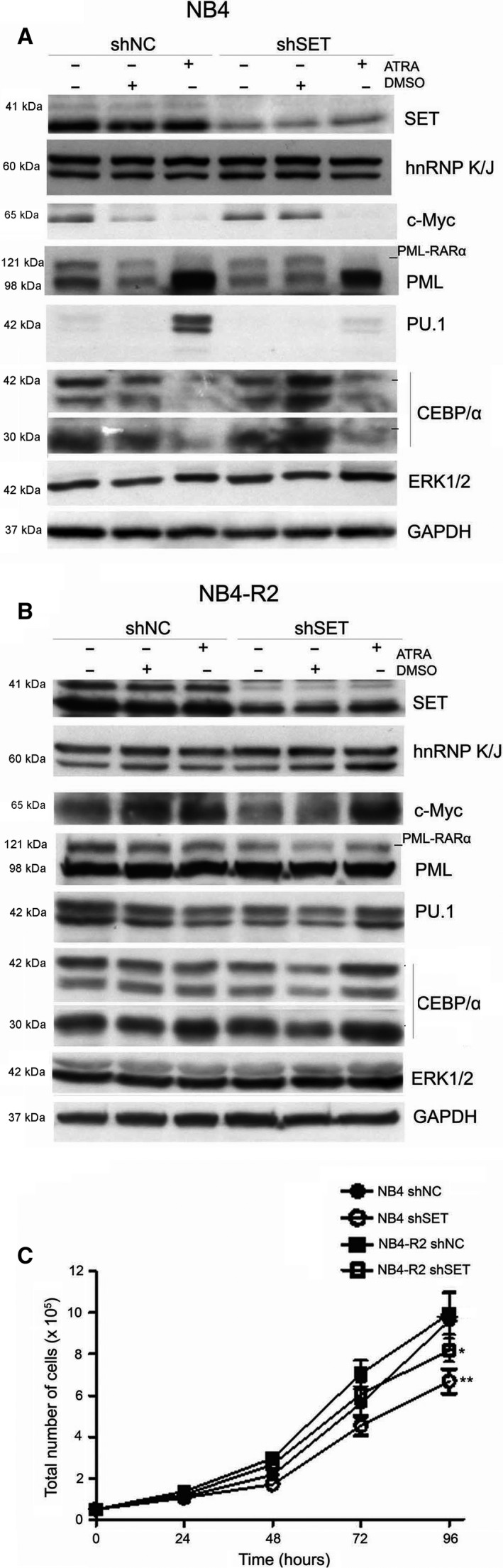
SET knockdown led to an alteration in the promyelocyte differentiation pathway and proliferation loss. Cells were transduced with shRNA to SET and selected with 0.5 µg·mL^−1^ puromycin for 15 days. (A) After establishing shSET NB4 and (B) shSET NB4‐R2, cell lines were treated with ATRA (1 µm) as previously described. The proteins were analyzed by using antibodies for SET, hnRNP K, c‐Myc, PML/PML‐RARa, PU.1, C/EBPα, and ERK1/2. GAPDH was used as a loading control. (C) For the growth curve, 5 × 10^4^ cells·mL^−1^ were seeded into a 24‐well plate, in triplicate, and counting was performed every 24 h for 96 h using the Trypan Blue exclusion method. Data are reported as mean ± SD of two independent experiments performed in triplicate. Statistical analysis was performed by one‐way ANOVA followed by the Bonferroni post‐test, *(*P* < 0.05), **(*P* < 0.01).

To understand how SET protein level participates in ATRA resistance and its relationship with hnRNP K in APL, we analyzed by immunoblotting hnRNP K and other proteins involved in promyelocyte differentiation. ShSET in association with ATRA showed lower level of PML and PML‐RARα proteins in NB4 cells and lower PML‐RARα in NB4‐R2 cells compared with shNC (Fig. 7A,B). Surprisingly, shSET NB4 cells under ATRA treatment showed a lower level of PU.1 protein compared with sh NC cells, suggesting a potential function of SET in PU.1 expression (Fig. 7A). Moreover, NB4‐R2 cells with shSET showed a reduction in C/EBPα and c‐myc levels compared with sh NC cells, suggesting a SET function in granulopoiesis and proliferation. In addition, NB4‐R2 shSET cells showed higher levels of PU.1 and C/EBPα under ATRA induction (Fig. 7B). Finally, we analyzed proliferation in shSET APL cells and both cells reduced proliferation (Fig. 7C).

## Discussion

This study is the first to demonstrate both the hnRNP K and SET profiles in the same APL patients and in cell lines simultaneously, as well as their involvement in ATRA‐induced differentiation of leukemic promyelocytes and to propose an ERK inhibitor combined with ATO as a potential new therapy for APL patients.

The accumulation of hnRNP K and SET mRNA in APL patient samples, during the diagnostic phase, in addition to the reduction in hnRNP K and SET during the chemotherapy phase, suggests not only the involvement of both genes in the leukemogenesis of APL, but also that hnRNP K and SET could be useful as prognostic indicators or treatment response biomarkers.

hnRNP K and SET levels were reduced in all patients during IP and MP, except for patient P5 who showed a relapse and higher levels than those detected during DP, mainly hnRNP K, associated with the molecular recurrence of PML‐RARα. According to previous reports, increased gene expression and protein levels of hnRNP K and SET are frequent events in several types of cancer and they have been proposed to be prognostic markers in leukemia, mainly in CML, increasing during a blast crisis [5, 6, 22, 23, 35]. A correlation of mRNA hnRNP K and SET levels in patients' samples during distinct APL phases suggests crosstalk between these genes in APL. Colocalization of hnRNP K with SET protein was detected by immunofluorescence microscopy in NB4 and NB4‐R2, but it disappeared during cellular differentiation induced by ATRA, supporting a connection between them during leukemogenesis in APL. Crosstalk between hnRNP K and SET in HNSCC was previously demonstrated by our group [36]. Specifically, SET accumulation in HNSCC regulates and interacts with hnRNP K, increasing its binding to nucleic acids, as demonstrated for Bcl‐xS, an anti‐apoptotic gene [11].

High levels of hnRNP K and SET in APL samples indicate that these proteins participate in the granulopoiesis process. hnRNP K has been reported to be crucial to embryogenesis in mice [36] and to be associated with several functions from gene expression regulation to protein translation [1]. Furthermore, hematopoietic stem cells from haploinsufficient animals are able to form hematological neoplasms in transplanted mice, suggesting hnRNP K as a tumor suppressor [36]. However, a previous work showed that hnRNP K overexpression is related to the progression of CML and proposed the protein as a potential therapeutic target [5]. Indeed, the role of hnRNP K in diverse cell cancer types is more complex and needs to be defined.

Similarly, SET involvement in hematopoiesis is unknown, except for the fact that SET is regulated by transcription factors involved in hematopoietic differentiation in AML, such as RUNX1 and GATA‐2 [37]. Since the expression of hnRNP K and SET was higher in APL patients, the knockdown of hnRNP K and SET proteins in APL cells was adopted as our model to study ATRA‐induced differentiation.

The hnRNP K knockdown in NB4 and NB4‐R2 cells reduced c‐Myc protein and proliferation. In this regard, it has been reported that hnRNP K regulates the activation of transcription and translation of c‐Myc [38, 39], which is directly involved in cell proliferation and cell cycle progression [40], and it is a potential therapeutic target in AML via apoptosis induction [41]. The cell death induced by hnRNP K knockdown in NB4 and NB4‐R2 cells is promoted at least in part by the reduction in Bcl‐xL protein (anti‐apoptotic) and procaspase‐3, suggesting apoptosis induction [42]. Moreover, another finding associated with cell death was the cleavage of SET protein in ~37, ~ 28, ~ 20, and ~ 18 kDa. Similar SET fragmentation into ~ 28 kDa and ~22 kDa and ~ 20 kDa has already been reported and is associated with apoptosis [17, 43].

Remarkably, the selection of APL cells with shRNA for hnRNP K using a low concentration of puromycin efficiently reduced hnRNP K levels accompanied by cell death, an effect amplified mainly in NB4‐R2 cells by treatment with ATRA. The decrease in cell viability was accompanied by a significant increase in cell differentiation, indicating that hnRNP K knockdown sensitizes both ATRA‐sensitive and ATRA‐resistant cells. We propose that cell differentiation occurred, at least partly, by the decrease in PML‐RARα oncoprotein and HDAC1 corepressor. The decrease in PML‐RARα levels is a critical event to restore the transcription of RARα target genes [44]. During granulocyte differentiation, C/EBPα levels decrease in the early stages and PU.1 levels increase in the late stages under ATRA treatment [45]. Our results showed a decrease in both proteins when hnRNPK protein is knocked down. The decrease in PU.1 can be explained by the fact that hnRNP K interacts with PU.1 during granulocytic differentiation induced by ATRA [46]. Therefore, in hnRNP K knockdown cells, the differentiation is activated by an alternative pathway.

This novel finding stimulated us to test a MEK1/2 inhibitor (U0126) to block hnRNP K phosphorylation. U0126 reduced hnRNP K activity and promoted cell death in NB4 and NB4‐R2 cells. Strikingly, this effect was accompanied by the cleavage of SET protein. These observations suggest an effect of U0126 similar to hnRNP K knockdown in APL cells, especially in cell viability. A correlation of hnRNP K and MAPK levels has been reported in CML, suggesting that ERK may have therapeutic potential [10, 47]. Based on the results obtained with the xenograft NB4‐R2 tumor model, we propose that the association of U0126 with ATO could be an effective therapeutic strategy in patients with ATRA resistance.

Interestingly, SET knockdown in both cells showed a lower decrease in proliferation when compared to hnRNP K knockdown (sh K), supporting our hypothesis that loss of viability in sh K cells occurs by SET cleavage rather than by the reduction in SET protein. SET protein has been reported to be overexpressed in leukemia and other cancer types and associated with cell proliferation and survival [21, 22, 48, 49]. Also, low cell proliferation in shSET cells was accompanied by a reduction in c‐Myc, which in turn is directly involved in cell cycle proliferation and progression [40]. Another new finding is ATRA response in NB4 shSET cells was accompanied by decreased PML‐RARα, PU.1, and C/EBPα levels. Therefore, hnRNP K protein depends on SET protein, so that a decrease in SET implies a decrease in hnRNP K activity as evidenced by PU.1 reduction in shSET cells. These results need to be explored in the future to understand the mechanism by which SET and hnRNP K are regulating PU.1.

In conclusion, we propose that (a) hnRNP K is a negative regulator of promyelocytic differentiation in APL by maintaining the stability of PML‐RARα, (b) this contributes positively to cell survival and maintenance through regulation of c‐Myc, Bcl‐xL, and SET proteins, (c) ERK signaling is essential for leukemogenesis and viability, by regulating hnRNP K protein (demonstrated by the treatment with U0126), and (d) hnRNP K and SET are regulating PU.1 expression/levels. Besides, because the inhibition of ERK signaling was associated with positive regulation of SET cleavage, we proposed the ERK/hnRNPK/SET signaling as target for APL and ATRA‐induced differentiation. Further studies will be needed to assess the full mechanism of hnRNP K and SET in granulopoiesis and to examine their potential as molecular markers in APL patients.

## Material and methods

### Patients

Samples of seven patients (three women and four men) with a confirmed diagnosis of APL by qRT‐PCR for PML/RARA and submitted to IC‐APL protocol treatment [50] were used. Samples were obtained at diagnosis, after induction, and during maintenance treatment. BM from APL patients and healthy donors (*n* = 2) was obtained from the biorepository of the Hematology Laboratory (the Ethical Committee for Human Research approved the protocol number: Hospital da Clínicas de Ribeirão Preto/HCRP#7147/2005), University Hospital of the Medical School of Ribeirão Preto, University of São Paulo (HCFMRP‐USP). BM (BM; *n* = 2) from healthy donors was used as control. Biological samples from all individuals were collected after all patients, and healthy donors signed written informed consent form. The study was approved by the Committee for Ethics in Research on Human of the School of Pharmaceutical Sciences of Ribeirão Preto, University of São Paulo, Ribeirão Preto, Brazil (Ethical approved protocol number 1.255.749). The work complies with the Code of Ethics of the World Medical Association (Declaration of Helsinki; http://www.cirp.org/library/ethics/helsinki/).

### Cell lines and culture conditions

The APL cells NB4 and NB4‐R2 t(15,17), respectively, responsive and resistant to ATRA‐induced differentiation therapy, were cultured in RPMI‐1640 medium supplemented with 10% FBS (GIBCO, Thermo Fisher Scientific Inc., Waltham, MA, USA), 1% antibiotic + antimycotic (Sigma‐Aldrich), in an incubator with a 5% CO_2_ humidified atmosphere, at 37 °C. NB4 and NB4‐R2 cells were periodically treated with ATRA to assess their ability to differentiate (data not shown). The karyotype of NB4 and NB4‐R2 cells confirmed the presence of t(15,17) (data not shown). HL‐60 and KG‐1a (American Type Culture Collection, Manassas, VA, USA), which did not present t(15,17), were cultured under the same conditions, but in Iscove's modified Dulbecco's medium in the presence of 20% FBS.

### Reagents

ATRA, monoethanolate‐specific inhibitor of MEK1 and MEK2 (U0126), propidium iodide (PI), Histopaque®‐1119 and 1077, puromycin, and DMSO were purchased from Sigma‐Aldrich. DMSO was used as a vehicle in ATRA‐induced differentiation and the U0126 experiments.

### Induction of differentiation in APL cells by ATRA

Treatment was performed with 1 µm ATRA (SIGMA‐Aldrich) applied to 2.5 × 10^5^ cells·mL^−1^ for 96 h, and the rate of differentiation was measured by determining the number of cells labeled with CD11b conjugated to PE (BD Company, Franklin Lakes, NJ, USA) by flow cytometry. The assays were performed in triplicate.

### RNA extraction and quantitative real‐time PCR

RNA was isolated from both IC‐APL (International Consortium on APL) samples and cell lines using TRIzol reagent (Invitrogen, Carlsbad, CA, USA) according to manufacturer's instructions. RNA integrity was determined with a 2100 Bioanalyzer (Agilent, Waldbronn, Germany) and quantified with a UV/VIS NanoDrop 1000 spectrophotometer (Thermo Fisher Scientific, Wilmington, DE, USA). c‐DNA synthesis was obtained using 500 ng RNA with the Go Script™ Reverse Transcription System (Promega, Madison, WI, USA). The PCR Master Mix reaction was performed using GoTaq® qPCR Master Mix (Promega). The oligonucleotides used for mRNA sequences from *HNRNPK*, *SET*, and *β‐GLOBIN* genes and conditions are described in Table S1. Relative changes in gene expression from qRT‐PCR experiments were calculated using the 2^−ΔΔCT^ (2^−[(CT sample − CT sample housekeeping gene) − (CT calibrator − CT calibrator housekeeping gene)]^) method [51].

### Immunoblotting assay and antibodies

The method is described as previously [24], and the antibodies were described in Table S2.

### Immunofluorescence analysis

Immunofluorescence was performed as previously reported [24] by using primary antibodies against hnRNP K and SET proteins (Table S2). Anti‐goat conjugated with DyLight®680 or an anti‐mouse conjugated with Alexa 488 (Invitrogen). Nuclei were stained with DAPI. The digital images were obtained using a Leica TCS SP8 confocal laser scanning microscope (Leica Microsystem, Wetzlar, Alemanha) using the 63× objective with 3× digital zoom. The scale bar was defined as 20 μm for images with 3× digital zoom.

### Assessment of cell viability and differentiation by flow cytometry

Myeloid cell differentiation was determined in NB4 and NB4‐R2 cells using the anti‐CD11b PE (BD) and flow cytometry. Cell viability was assessed by PI (1 µg·mL^−1^) staining and flow cytometry (BD FACSCalibur, San Jose, CA, USA). All assays were performed in triplicate.

### Plasmids and interference RNA

Knockdown of hnRNP K or SET proteins was performed in NB4 and NB4‐R2 cells using the MISSION short‐hairpin RNA (shRNA) plasmid (Sigma‐Aldrich). After screening using five shRNA sequences for the hnRNP K protein in APL cells, we selected for the assay a clone with the highest efficiency for the knockdown of the target protein (data not shown). The selected shRNA sequences were two nonmammalian targets as NC (pLKO.1puro, SHC202, and SHC002; SIGMA), human hnRNP K (sh3, clone 3‐NM _002140.3; SIGMA), and human SET (shSET, NM_003011.1‐467s1c1; SIGMA, TRCN0000063717). NB4 and NB4‐R2 cells were transduced with the lentiviral particles constructed with the ViraPower™ Lentiviral Expression System (MISSON® Lentiviral Packaging Mix‐Invitrogen) and HEK293FT (Life Tech, Thermo Fisher Scientific) according to the manufacturer's protocol. After selection with 500 ng·mL^−1^ puromycin, the extent of knockdown of hnRNPK and SET proteins in both APL cell lines was determined by immunoblotting.

### Cell growth curve

According to the indicated time and conditions in each assay, the cell growth curve was constructed using the Trypan Blue exclusion assay.

### Panoptic staining

For fast panoptic staining, 1 × 10^4^ cells were washed in PBS 1× and spin down on a microscope slide by centrifugation at 200 ***g*** for 1 min at room temperature. The cells were stained following the manufacture's protocol (Laborclin, Pinhais, Paraná, Brazil). Briefly, the cells were incubated for 5 s in solutions #1, #2, and #3 and subsequently washed with deionized water. Microscopy analyses were performed in a brightfield, PlasDIC, phase contrast, and fluorescence AXIOVERT 40 CFL (ZEISS, Gotting, Germany) at 400× original magnification.

### Xenograft tumor model using NB4‐R2 cells and immunohistochemistry

A human NB4‐R2 xenograft tumor model in athymic nude mice (Balb/C) was used as previously described [24]. NB4‐R2 wild‐type cells (5 × 10^6^) were transplanted to induce the tumors bilaterally. The animal experimentation was approved and conducted according to the ethical conduct outlined in the Care and Use of Animals for Experimentation of the School of Pharmaceutical Sciences of Ribeirão Preto, University of São Paulo (protocol number no 16.1.90.60.0). At autopsy, tumors were weighed and fixed in 4% neutral buffered formalin for 24 h and then in 70% ethanol until the beginning of histological processing. Six‐micrometer tissue sections were obtained. Staining by H&E was performed and analyzed under a light field microscope at 100× magnification. For immunohistochemistry, antigen recovery was performed in a microwave oven for 30 min with citrate buffer. Blocking of nonspecific sites was performed with PBS‐T + 3% BSA for 2 h at room temperature. Endogenous peroxidase blockade was done with 3% hydrogen peroxide for 15 min. Slides were incubated with Ki67 primary antibody (Abcam, Cambridge, MA, USA, ab15580, 1 : 100 in PBS‐T + 3% BSA) for 16 h at 4 °C in a humid chamber. LSAB (Dako Denmark A/S, Agilent Technologies Inc., Santa Clara, CA, USA) was used as the universal secondary antibody, and DAB was used as the chromogen. The tissue samples were counterstained by immersion in Harry's hematoxylin.

### Statistical analysis

Statistical analysis was performed using graphpad prism software version 5.0 (GraphPad Software Inc., La Jolla, CA, USA). The tests used are indicated when each result is reported.

## Conflict of interest

The authors declare no conflict of interest.

## Author contributions

KSP, EMR, and AML conceived and designed the project; KSP, RNG, LBF, CBG, MSB, and VMA acquired the data; KSP, EMR, and AML analyzed and interpreted the data; KSP and AML wrote the paper; LJG, EMR, and AML revised the paper.

## Supporting information


**Table S1**. The oligonucleotides (primers) used for mRNA sequences in qRT‐PCR assays.
**Table S2**. Antibodies used in the present study.

## Data Availability

All supporting data are included in the manuscript and as supplementary information.
