# Supplementary material for: Crosstalk between hnRNP K and SET in ATRA‐induced differentiation in acute promyelocytic leukemia
Source: FEBS Open Bio. 2021 Jun 17;11(7):2019–32. doi: 10.1002/2211-5463.13210 (PMC8255839; doi:10.1002/2211-5463.13210)
Supplement: Supplementary file 1 — Table S1. The oligonucleotides (primers) used for mRNA sequences in qRT‐PCR assays. Table S2. Antibodies used in the present study. [file FEB4-11-2019-s001.docx]

**Table S1. The oligonucleotides (primers) used for mRNA sequences in qRT-PCR assays.**

| *GENE* | *SEQUENCE (5’-3’)* | *NM* |
| --- | --- | --- |
| *β-GLOBIN* (F) | GCCTCGCTGTCCACCTTCCA | NM_00518.4 |
| *β-GLOBIN* (R) | AGAAAGGGTGTAACGCAACTAAG |  |
| *HNRNPK* (A) (F) | AGT ATG CAG ATG TTG AAG GAT T | NM_031263.2 |
| *HNRNPK* (B) (F) | TGT GAA GCA GTA TTC TGG AAA GT | NM_ 031262.2 |
| *HNRNPK* (A/B) (R) | GCA GAA CAC CTA TGA AGC AGA G |  |
| *SET* (1) (F) | TCCCGCCTCAAAAGAAGAAACCAA | NM_001122821.1 |
| *SET* (2) (R) | GCCAAAGTCAGTAAAAAGGAGC | NM_003011.3 |
| *SET* (1/2) (R) | AAATCTCCTCACTGGCTTGTTCAT |  |
| qRT-PCR cycling conditions | | |
| 2 min at 95ºC, followed by 40 cycles of 15 s at 95ºC, 30 s at 55ºC, except for *HNRNPK* for which the conditions were 20 s at 72ºC and 2 min at 95ºC, followed by 40 cycles of 15 s at 95ºC, 1 min at 60ºC, and 15 s at 95ºC. | | |

*β-GLOBIN* was used as the housekeeping gene. Forward primer (F). Reverse primer (R). *HNRNP K isoform a (A), HNRNP K isoform b (B), SET isoform 1 (1), SET isoform 2 (2).*

**Table S2. Antibodies used in the present study.**

| **Antibody** | **Code** | **Origin** | **Dilution** | **Manufacturer** |
| --- | --- | --- | --- | --- |
| hnRNP K | R8903 | mouse | 1:100,000 | Sigma Aldrich |
| MAPK | M5670 | Rabbit | 1:10,000 | Sigma Aldrich |
| SET | SAB4200479 | Mouse | 1:50,000 | Sigma Aldrich |
| SET | sc-5655 | Goat | 1:2,000 | Santa Cruz |
| PML | sc-5621 | Rabbit | 1:1,000 | Santa Cruz |
| Procaspase-3 | #9665 | Rabbit | 1:1,000 | Cell Signaling |
| c-Myc | #5605 | Rabbit | 1:1,000 | Cell Signaling |
| C/EBPα | #9197 | Rabbit | 1:1,000 | Cell Signaling |
| GAPDH | #2592 | Rabbit | 1:10,000 | Cell Signaling |
| HDAC1 | #2062 | Rabbit | 1:1,000 | Cell Signaling |
| STAT3 | #9139 | Rabbit | 1:1,000 | Cell Signaling |
| PU.1 | ab76543 | Rabbit | 1:1,000 | Abcam |
| Histone H3 | #9715 | Rabbit | 1:5,000 | Cell Signaling |
| Β-actin | sc-47778 | Mouse | 1:5,000 | Santa Cruz  Biotechnology |
| mouse | 04-18-06 | Goat | 1:50,000 | KPL |
| rabbit | 65-6120 | Goat | 1:10,000 | Invitrogen |
| goat | 14-13-06 | Rabbit | 1:5,000 | KPL |
|  |  |  |  |  |
